# The empirical effect of agricultural social services on pesticide inputs

**DOI:** 10.1038/s41598-024-67016-7

**Published:** 2024-07-10

**Authors:** Hui Na, Xiumei Yan, Rui Xing, Anyin Jiang

**Affiliations:** 1 School of Economics, Lanzhou University, Lanzhou, 730000 China; 2 Gansu Agricultural Mechanization Technology Extension Station, Lanzhou, 730000 China; 3 School of Economics, North Minzu University, Yinchuan, 750021 China

**Keywords:** Agricultural social services, Pesticide inputs, Agricultural green development, China, Ecology, Environmental social sciences

## Abstract

Agricultural social services (ASS) play an important role in improving the efficiency of agricultural operations, reducing agricultural production costs, and promoting sustainable agricultural development. Using data from the 2020 China Rural Revitalization Survey, this study analyzes the impact of ASS on reducing pesticide inputs. The results show: (1) ASS play a significantly positive role in reducing pesticide inputs. (2) Heterogeneity analyses show that ASS’ role in reducing pesticide inputs is stronger for farming households with small farms, which participate in cooperatives, and do not have members involved in non-farm employment than that for farming households with large farms, which do not participate in cooperatives, and have members involved in non-farm employment. (3) Mechanism analysis shows that ASS’ green perception and demonstration-led effects contribute to reducing pesticide inputs by 148.6% and 36.8%, respectively, at the 1% level. Finally, this study proposes relevant policy recommendations for promoting ASS, promoting the continuous operation of farmland, and encouraging farmers to participate in ASS.

## Introduction

As modern input in agricultural production, pesticides play an important role in preventing and controlling crop pests and diseases, thereby increasing food production and ensuring food supply^1–3^. However, their irrational use can negatively affect the quality of agricultural products and the ecological environment^4^. According to the Food and Agriculture Organization (FAO), the global pesticide use in 2020 was 2661.124 billion tonnes, while a around tenth of it was used in China at 273.376 billion tonnes. Moreover, China’s average pesticide use per hectare of cropland is 1.95 kg, higher than the world average of 1.81 kg^5^. As one of the largest producer and consumer of pesticides in China, excessive pesticide use by farmers has aggravated agroecological pollution, reduced the quality of agricultural products, and threatened human health^6^. Promoting the reduction in pesticide use is essential for strengthening food security and accelerating the overall green agricultural tranformation in China, as well as an important measure for guaranteeing food quality and fostering sustainable development.

As the country’s environmental awareness has increased, the Chinese government has gradually increased its attention and efforts to address excessive pesticide use. The Ministry of Agriculture and Rural Affairs has formulated an action program for reducing chemical pesticides by 2025, which includes reducing the intensity of pesticide use in rice, wheat, corn and other major food crops by 5%. However, due to the dispersed, hidden, and lagging nature of agricultural surface pollution, relying solely on the policy to control the pesticide use may not be effective^7^. Rather, encouraging farmers to take the initiative to reduce pesticide use is key for solving agricultural surface pollution.

Farmers, as economically rational persons, are driven by the pursuit agricultural operation efficiency to increase pesticide use to reduce the risk of crop yield reduction^8^. Alternatively, they may be reluctant to adopt highly efficient and low-toxic pesticides because of higher switching costs, or are inhibited from adopting green prevention and control technologies considering the potential business risks. Indeed, pesticide use has become a major topic in the study of agricultural production behavior. Numerous studies have analyzed the pesticide use behavior of farmers in terms of their personal characteristics, household production characteristics, and external factors^9^. Farmers’ behavior characteristics analyzed by researchers include gender, age, risk attitude, and education level. Specifically, evidence shows that the higher the education level of farmers, the more likely it is that they will use pesticides according to the instructions such that they can reduce the amount of pesticides used^10,11^. Next, family characteristics include the family’s economic situation^12,13^, the scale of production and operation, and sales patterns. Empirical evidence shows that the higher the household income and stronger the economic power, the greater the likelihood that farmers will buy more expensive bio-pesticides, and the more likely they will use pesticides rationally^14^. Finally, external factors mainly include those at the level of market supply and demand, and pesticide production. Specifically, new agricultural operators can help regulate the behavior of farmers, and reduce the excessive use of highly toxic pesticides and insecticides. Further, pesticide sellers’ guidance on pesticide application techniques and explanation of pesticide ratios to farmers can influence their pesticide use^15–17^.

As the agricultural production process is labor intensive, farmers’ heterogenous characteristics can lead to differences in the judgement of pests and diseases, selection of pesticide types, operation of pesticide application equipment, and mastery of the application time^18,19^, among other factors. In particular, farmers’ profit-seeking behavior can easily induce excessive pesticide use. This can lead to pesticide surface pollution, endangering the agroecological environment, and the quality and safety of food products^20,21^. A potential solution can be ASS.

ASS can help quantify pesticide use through modern agricultural science and technology. It can provide farmers, especially small ones, with pest control programs, pesticide use ratios, and advanced pesticide use technology, thus reducing pesticide use^22,23^. Meanwhile, ASS can be used to maximize profits via enhancing operations by improving agricultural infrastructure, continuous farmland operation, agricultural machinery use, and thus, achieve large-scale operation. This can reduce the difficulty of adopting mechanized operations and pesticide reduction technology, and make using ASS to promote pesticide reduction practically feasible^24^.

Accordingly, understanding Chinese farmers’ ability and tendency to deal with goal conflict in pesticide use decision-making, influenced by ASS, and clarifying how it affects their pesticide use behaviors can help in finding an effective way for farmers to implement pesticide reduction behaviors on their own. To explore this, this study analyzes the impact of ASS on reducing pesticide inputs. Overall, the findings can help achieve the dual goals of green development of the planting and reducing agricultural surface pollution.

This study’s contributions are as follows: First, by exploring the pathway of reducing pesticide use via ASS, this study tries to provide an alternative to the unidirectional thinking of promoting agricultural green development. This study also reveals the underlying mechanism of the relationship between ASS and pesticide use reduction. These insights can be valuable for reducing pesticide use with the ASS. Second, this study clarifies the internal mechanism of reducing pesticide use from two perspectives: the green perception effect, and ASS’ demonstration-led effect. Thus, it reveals a new path of pesticide use reduction by farmers which can be leveraged to deepen the role of social services in the sustainable development of agriculture, and promote green agricultural transformation and development.

## Theoretical analysis and hypotheses

### Green perception effect of ASS

The theory of planned behavior posits that the intention of cognitive and attitude control together shape the individual’s behavioral intentions and behavior. Thus, the higher the level of green knowledge of farmers, the more rational they will be in agricultural decision-making. They will not only focus on the cost, income, and other issues, but also consider the harm of pesticides on the environment. Specifically, Chinese social service organizations can be involved in the promotion of pesticide reduction technology, such as new types of pesticides and biopesticides^25^. Further, the advantages of the new technologies can be publicized. This can help in subconsciously increasing the knowledge of farmers about green production and improve their ecological cognitive level, and thus, promote the reduction of pesticide inputs^26^. Moreover, compared with other organizations in China, socialized service organizations can regularly provide free technical guidance on green production, helping farmers understand and master the knowledge and skills of green agricultural production^27^. This can help increase the implementation of green production techniques among farmers, and thus, reduce pesticide inputs^2^. Meanwhile, by providing green agricultural products sales, certification, and other services to help small farmers understand the market demand for green products and advantages, ASS enhance their sense of identity for green products^28^. This can effectively promote pesticide use reduction by farmers, improve the quality and safety of agricultural products, meet consumers’ demand for green, healthy and environmentally friendly products, and promote the green and sustainable development of agriculture^29^. Accordingly, the first hypothesis is proposed as follows:

#### H1

ASS can increase green perceptions among farmers, and thus, reduce pesticide inputs.

### Demonstration-led effect of ASS

**Figure 1:**
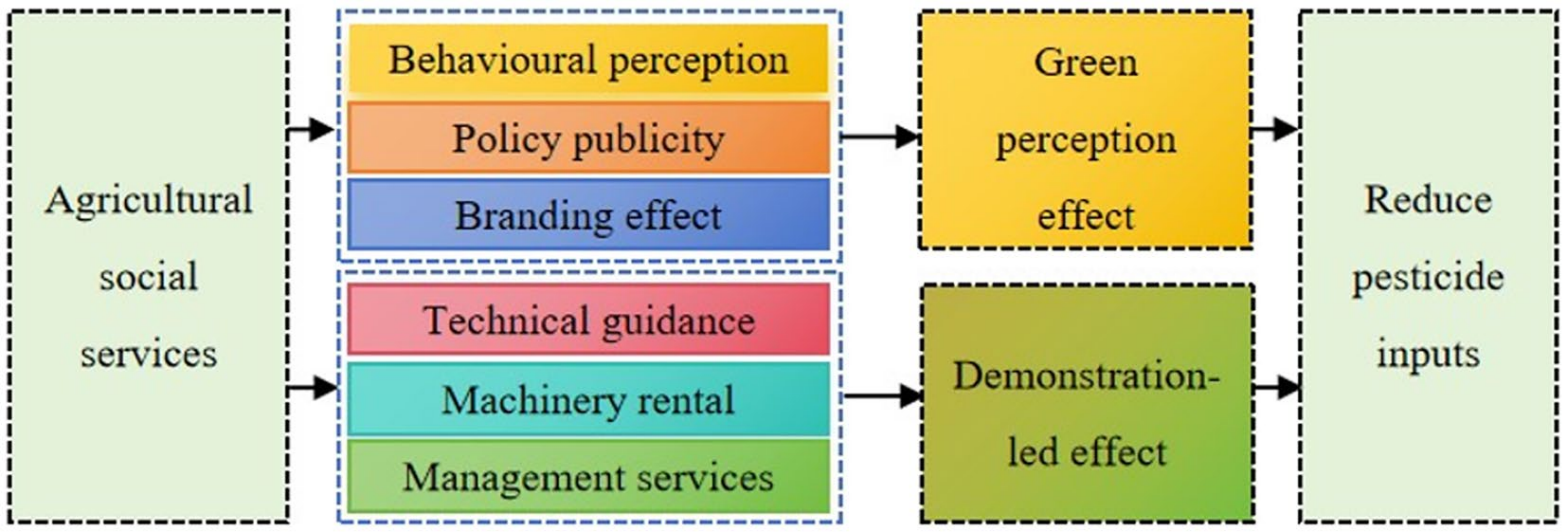
Logic schematic.

Regarding the demonstration-led effect, first, ASS help farmers master advanced agricultural production technology and management methods by providing professional agricultural technology, agricultural machinery, agricultural information^30^, etc. This helps enhance the production efficiency and technical level of farmers, and reduces the purchase of agricultural machinery and cost of pest control, and thus, reduces pesticide inputs^31^. Second, by providing pre-production, production, and post-production services, ASS help farmers obtain more market information and sales channels, gain a better understanding of market demand, adjust the planting structure, and leverage the brand effect to improve the competitiveness of their agricultural products in the market. Thus, besides themselves, other farmers will be also forced to consider the quality of agricultural products, and thus, reduce pesticide inputs to a certain extent^32^. In addition, the ultimate goal of ASS is to increase the efficiency of pesticide inputs through agricultural machinery and modern pesticides^33–35^, which in turn will increase crop yields and household incomes. Accordingly, the second hypothesis is proposed as follows (Fig. 1. shows the logic schematic):

#### H2

ASS play a demonstrative and leading role for farmers, which in turn reduces pesticide inputs.

## Data and methods

### Data

The data come from the 2020 China Rural Revitalization Survey (CRRS) data. CRRS considers the level of economic development and the basic situation of agricultural development in the eastern, central, western, and northeastern regions of China. It covers 10 provinces, 50 counties (cities), and 156 townships (towns) in China according to the equal spaced random sampling method of per capita GDP of each county (town). This study retains key variables such as the amount of pesticide inputs and socialized service expenditures of households, the age of the household head, education level, family size, farmland area, parcel number, and village characteristics. To improve the sample’s representative, samples where the cropland areas of the farmer’s household and their village is zero are excluded. This yields a final sample with 2948 observations from 10 provinces.

### Variables

#### Dependent variable

The dependent variable is the sum of pesticides, herbicides, and anti-pest drugs purchased by the sample households. Because this cost is a family agricultural operation expenditure, farmers have a clear memory of its amount. Pesticides play an important role in the control of agricultural pests on crops and increase crop yields. However, their overuse will cause the quality of agricultural products to decline, aggravate agricultural surface pollution, and endanger human health. Hence, the amount of pesticide inputs is appropriate as a dependent variable.

#### Independent variables

The independent variable is ASS. Specifically, households that purchased mechanical, drone, and cooperative provided spraying services in the spraying segment of the questionnaire were assigned a value of 1, and 0 otherwise.

#### Control variables

To avoid the omission of variables leading to model estimation bias, this study includes variables such as household head, family, farmland, and village characteristics. The household head plays an important role in making agricultural operation decisions for the family and through the coordinated allocation of family resources, thereby improving the efficiency of family resource use. Family characteristics include family labor structure, operation concepts, and capital accumulation. Village characteristics influence household development. The variables are as shown in Tables 1 and 2.

**Table 1:** Variable definitions.

Variables	Definitions
Pesticide inputs	Total expenditure by farmers on pesticides, herbicides and anti-pest drugs (yuan^b^)^a^
ASS	Whether farmers purchase agricultural social services: 1 = Yes; 0 = No
Age	Head of household’s age (years)
Education	Educational level of household head: 0 = Illiterate; 1 = Primary school; 2 = Junior high school; 3 = High school; 4 = Secondary school, 5 = Vocational high school, 6 = University college, 7 = Undergraduate college, 8 = Postgraduate school, 9 = Other
Family size	Total number of family members
Land size	How many acres of cropland does the household own? (mu = 0.0667 ha)^a^
Land parcels	How many parcels of farmland does the household own?
Land transfer	Does the household participate in the transfer of farmland? 1 = Yes; 0 = No
Non-farm	Does the household have any non-farm employed members? 1 = Yes; 0 = No
Labor time	How many days of agricultural work are done by the household? (day)^a^
Income	What is the total annual household income? (yuan^b^)^a^
Pesticide package	How do you dispose of pesticide packaging: 1 = Buried on site; 2 = Centralized landfill; 3 = Incineration; 4 = Recycled to fixed sites; 5 = Recycled to agricultural markets; 6 = Discarded; 7 = Other
Package hazards	What do you think are the hazards of pesticide packaging: 1 = Destroying the soil; 2 = Affecting crop yields; 3 = Polluting the environment; 4 = Other
Package recycling	What do you think is the resistance to recycling pesticide packaging: 1 = No subsidy, not willing to recycle; 2 = It doesn’t matter if you recycle or not; 3 = Neighbors don’t recycle; 4 = No penalties; 5 = Other
Perceived	Pesticide use per acre compared to 5 years ago: 1 = Decrease, 2 = No change, 3 = Increase
Distance	How many kilometres is the farmer from the town? (kilometer)
Village terrain	Terrain of the village where the farmer is located: 1 = Plain; 2 = Hilly; 3 = Mountain; 4 = Semi-mountains
Demonstration	Number of households in villages led?^a^

^a^The variables related to value are processed using a logarithm.

^b^Yuan is the Chinese currency: 1 USD = 6.89 yuan in 2019.

**Table 2:** Descriptive statistics and mean differences.

Variables	Purchase of ASS		No purchase of ASS		Mean-diff	t-value
	Mean	SD	Mean	SD		
Pesticide inputs	5.428	2.160	1.800	2.633	− 2.915***	− 26.781
Age	54.488	10.585	54.524	11.008	1.411***	3.254
Education	2.353	1.873	2.563	1.832	0.182**	2.440
Family size	3.106	1.522	3.250	1.536	0.233***	3.798
Land size	2.607	1.227	1.834	1.135	− 0.767***	− 15.954
Land parcels	9.642	7.830	7.117	11.505	− 3.779***	− 9.632
Land transfer	0.500	0.500	0.448	0.497	− 0.073***	− 3.635
Non-farm	1.830	1.278	2.111	1.562	0.193***	3.348
Labor time	4.304	1.492	3.763	2.118	− 0.408***	− 5.506
Income	10.395	1.819	10.513	1.917	− 0.005	− 0.068
Pesticide package	4.407	1.941	4.967	2.005	0.509***	6.386
Package hazards	3.344	0.875	3.497	0.762	0.086***	2.589
Package recycling	3.206	1.655	3.599	1.587	0.343***	5.234
Perceived	2.264	0.663	2.724	0.566	0.387***	15.168
Distance	5.761	5.782	5.913	5.370	− 0.686***	− 3.060
Village terrain	1.618	0.830	2.361	0.852	0.622***	17.683
Demonstration	3.827	2.449	3.909	2.463	0.239**	2.414

***, **, and * indicate signiffcance at the 1%, 5%, and 10% levels, respectively.

### Methods

The baseline model of ASS on pesticide inputs is as follows:

1 $$Pesticide_{i} = \beta_{0} + \beta_{1} Service_{i} + \beta_{2} Head_{i} + \beta_{3} Family_{i} + \beta_{4} Villages_{i} + \delta_{i}$$

$Pesticide_{i}$ denotes the amount of pesticide inputs by farmers, $Service_{i}$ denotes the expenditure of farmers on purchasing ASS, ${Head}_{i}$ denotes household head characteristics, ${Family}_{i}$ denotes household characteristics, ${Villages}_{i}$ denotes village characteristics. ${\beta }_{0},{ \beta }_{1}$*,*${\beta }_{2}$*,* ${\beta }_{3}$*,* and ${\beta }_{4}$ denote the respective coefficients to be estimated, and ${\delta }_{i}$ denotes the random error term.

The instrumental variable regression model is set up as follows:

2 $$Pesticide_{i} = \gamma_{0} + \gamma_{1} IV_{i} + \gamma_{2} Head_{i} + \gamma_{3} Family_{i} + \gamma_{4} Villages_{i} + \pi_{i}$$

${IV}_{i}$ denotes the instrumental variable for the independent variables (Number of village cooperatives) and the coefficients are set in the same way as in Eq. (1). The instrumental variables were selected for the following reasons: because village cooperatives do not directly affect the purchase of ASS by farmers and satisfy the exogeneity requirement of the instrumental variable. At the same time, village cooperatives, as the main providers of ASS, are able to provide farmers with agricultural machinery rental services and related technical guidance, which satisfies the relevance of the instrumental variable.

Next, based on Eq. (1), the change in the average pesticide inputs per mu compared with five years ago and number of farmers in the lead villages are used as moderators. Specifically, their interaction terms with the independent variables are used to indicate the green perception and demonstration-led effects, respectively. The following model was used:

3 $$Pesticide_{i} = \rho_{0} + \rho_{1} Mechanism_{i} + \rho_{2} Head_{i} + \rho_{3} Family_{i} + \rho_{4} Villages_{i} + \omega_{i}$$

${\text{Mechanism}}_{{\text{i}}}$ denotes the cross-multiplier term between ASS and the moderating variables, and the other variables and coefficient settings are the same as those in Eq. (1).

Note that all experimental protocols have been approved by the School of Economics, Lanzhou University, Gansu Agricultural Mechanization Technology Extension Station, and the School of Economics, North Minzu University. All experiments were conducted in accordance with relevant guidelines and regulations.

### Consent to participate

I am free to contact any of the people involved in the research to seek further clarification and information.

## Results

### Basic regression analysis

For the stability of the regression results, the analyzes were conducted through two models: ordinary least squares (OLS) and Tobit. As shown in Table 3, ASS play a positive and significant role in reducing pesticide inputs. Household head’s education level, land size, land parcel number, labor time, and distance from the town all play a positive and significant role in reducing the amount of pesticide inputs. The reasons may be as follows: when the household head is more educated, they are more likely to adopt new agricultural technology to improve the efficiency of pesticide use and their knowledge of the environment can be higher than that of other less educated farmers. When land size and land parcels are larger, it provides a convenient condition for the mechanization of agricultural production. Farmers tend to centralize their operations to reduce the unreasonable problems caused by manual application of pesticides, and thus, helps reduce pesticide inputs. The longer the agricultural labor time, the more farmers will mitigate pests and diseases by improving field management, thus reducing pesticide inputs. In contrast, town-based agricultural procurement centers determine to some extent the spraying behavior of farmers, especially when the degree of convenience is high. Pesticide sellers often give chemical agricultural purchasers an introduction to the performance and type, helping ensure that farmers are well informed and can reduce pesticide inputs while improving the efficiency of pesticide use.

**Table 3:** Regression results of ASS on pesticide inputs.

Variables	Tobit	
	Coeff	Std.
ASS	3.416***	0.134
Age	− 0.001	0.005
Education	0.076**	0.030
Family size	0.037	0.037
Land size	0.527***	0.058
Land parcels	0.061***	0.006
Land transfer	0.161	0.147
Non-farm	− 0.033	0.045
Labor time	0.119***	0.037
Income	− 0.084***	0.030
Pesticide package	− 0.249***	0.029
Package hazards	− 0.077	0.067
Package recycling	− 0.213***	0.035
Perceived	− 1.842**	0.087
Distance	0.018*	0.010
Village terrain	− 0.130*	0.070
_cons	6.450***	0.658
*R*^2^	0.308	
*N*	2948	

***, **, and * indicate signiffcance at the 1%, 5%, and 10% levels, respectively.

Conversely, total household income, pesticide packaging, pesticide hazards, package recycling, perceived use, and village terrain play a negative and significant role in reducing pesticide inputs. This may be because when household income is higher, households are driven by profit-seeking behaviors and are more willing to purchase higher-priced biopesticides, which in turn is not conducive to reducing pesticide inputs. Further, due to the prominent problems of rural hollowing out and aging, the perception of protecting the agroecological environment is low. Hence, the perceptions of spraying per mu cannot reduce the pesticide inputs. Additionally, the perception of disposal of pesticide packages, hazardous level of pesticides, and recycling of pesticide packages are also low, resulting in their negative impact in reducing pesticide inputs. The village terrain determines the degree of agricultural mechanization to some extent. Thus, compared to the plains, villages in other terrains have a lower degree of spraying with the help of mechanical spraying and most mainly spray manually. This is not conducive to reducing pesticide inputs.

### Robustness test

#### Endogeneity problem

Table 4 shows that the Cragg–Donald Wald F-statistics for the first stage are all greater than 16.38 at the 10% level of bias, indicating the absence of weak instrumental variables. Further, the *p*-value of the Kleibergen–Paap LM statistic is less than 0.01, which satisfies the correlation requirement for instrumental variables^36^. ASS play a positive and significant role in using pesticide inputs in the two-stage least squares (2SLS) regression at the 1% level, and its estimated coefficient on the amount of pesticide inputs is 6.495. Moreover, the regression results of the IV-Tobit model are even stronger.

**Table 4:** Endogeneity regression results.

Variables	2SLS		IV-Tobit	
	Coeff	Std.	Coeff	Std.
ASS	6.495***	0.771	13.735***	0.936
Other Variables	Yes	Yes	Yes	Yes
_cons	0.007	1.247	− 8.330*	4.339
Cragg–Donald Wald F statistic	27.710			
Kleibergen–Paap rk LM statistic	85.095***			
R^2^	0.230		0.311	
N	2948		2948	

***, **, and * indicate signiffcance at the 1%, 5%, and 10% levels, respectively.

#### Propensity score matching

The purchase of ASS by farmers is subject to many internal and external factors, which may lead to sample selectivity bias. The propensity score matching (PSM) method can help reduce this bias by making samples as close as possible to the random experiment data through matching and re-sampling. Here, the PSM model set the farmers who purchased ASS as the treatment group (Treated), and those who did not purchase as the control group (Control). As the treated group did not purchase ASS, which are not measurable for reducing pesticide use, the characteristics of the control group of farmers were modelled through various matching methods. Specifically, the PSM model derives the average treatment effect (ATT) of ASS on reducing pesticide inputs by constructing a counterfactual framework. The following model was constructed based on Eq. (1):

4 $$ATT = E(Pesticide_{i1} |D_{i} = 1) - (Pesticide_{i0} |D_{i} = 1) = E(Pesticide_{i1} - Pesticide_{i0} |D_{i} = 1)$$

${Pesticide}_{i1}$ denotes the pesticide inputs with purchase of ASS, ${Pesticide}_{i0}$ denotes the pesticide inputs without purchase of ASS, and ${D}_{i}=1$ is restricting the study sample to the group of purchase of ASS.

####  Balance test

To examine whether PSM better balances the original sample data, a balance test is needed. As seen from Table 5, the absolute value of standardized deviation of most variables after matching is less than 20% compared with that before matching. Further, the t-test results of most variables after matching do not reject the original hypothesis of no systematic difference between the treated and control groups (*P* > 0.05), thus indicating that the PSM results passed the balance test and the quality of matching is better. Due to space limitations, Table 5 only exhibits the results of nearest-neighbor matching.

**Table 5:** Balance test results.

Variables	Pre-match mean		Post-match mean		Bias (%)		Post-match t-tests	
	Treated	Control	Treated	Control	Unmatched	Matched	T-value	*P*-value
Age	54.488	54.525	54.481	53.517	− 0.3	8.9	2.470	0.014
Education	2.352	2.549	2.351	2.335	− 10.6	0.9	0.250	0.803
Family size	3.106	3.237	3.107	3.221	− 8.6	− 7.5	− 2.090	0.037
Land size	2.606	1.852	2.605	2.877	64.3	− 23.2	− 5.490	0.000
Land parcels	9.641	7.196	9.643	12.588	24.7	− 29.8	− 4.580	0.000
Land transfer	0.500	0.449	0.500	0.524	10.1	− 4.9	− 1.350	0.177
Non-farm	1.830	2.095	1.830	1.822	− 18.7	0.5	0.150	0.878
Labor time	4.303	3.804	4.303	4.406	27.5	− 5.7	− 1.790	0.074
Income	10.395	10.505	10.401	10.523	− 5.9	− 6.5	− 1.700	0.089
Pesticide package	4.406	4.941	4.405	4.337	− 27.1	3.4	0.970	0.334
Package hazards	3.344	3.490	3.343	3.332	− 17.8	1.3	0.340	0.733
Package recycling	3.205	3.598	3.203	3.282	− 24.2	− 4.8	− 1.300	0.192
Perceived	2.264	2.720	2.263	2.256	− 73.9	1.1	0.270	0.790
Distance	5.761	5.942	5.760	5.749	− 3.2	0.2	0.060	0.954
Village terrain	1.618	2.340	1.616	1.762	− 86.5	− 17.4	− 4.770	0.000

#### Kernel density test

**Figure 2:**
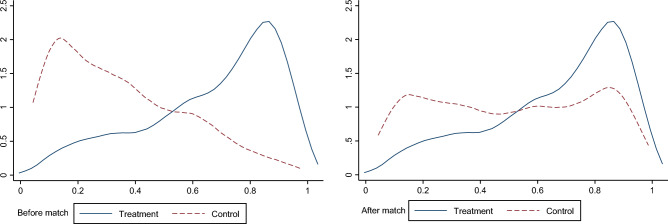
Changes in the treated and control groups before and after matching.

The kernel density graph is a fundamental test to examine the effectiveness of PSM as different matching methods have different sample loss amounts. This study simultaneously uses four methods, namely, nearest neighbor matching, caliper matching, spline matching and kernel matching, to demonstrate the matching effect. Due to space limitations, this study only reports the kernel density graph of the common support domain of the nearest neighbor matching. As seen in Fig. 2, compared with the pre-matching, the two lines of the treatment and control groups are closer after matching, which indicates that the matching effect is better.

Table 6 shows that ASS has a significantly positive effect on the reduction of pesticide inputs. Further, the four matching methods’ results are relatively close, indicating the robustness of the estimation results. The mean value of the average treatment effect is 1.632. That is, if farmers who purchase ASS hypothetically did not purchase it, the average proportion of pesticide input reduction is 3.823; however, if they purchase ASS, this proportion rises to 5.423, which is an increase of 1.6 (close to the mean average treatment effect). This suggests ASS positive effect on reducing pesticide inputs.

**Table 6:** PSM regression results.

Matching methods	Treated	Controls	ATT
Nearest neighbour matching	5.423	3.934	1.614***(0.172)
Caliper matching	5.423	3.768	1.655***(0.155)
Spline matching	5.423	3.820	1.604***(0.142)
Kernel matching	5.423	3.768	1.655***(0.155)
Average	5.423	3.823	1.632

***, **, and * indicate signiffcance at the 1%, 5%, and 10% levels, respectively.

### Heterogeneity analyses

#### Size of operations

The median cropland area is used to divide the sample into large and small area groups. The results in Table 7 show that farmers in the small area group are usually more likely to achieve precision in pesticide use. This may be because farmers can precisely determine the type, dosage, and timing of pesticide use based on the specific occurrence of pests and diseases, and the needs of crop growth. This kind of precision use helps reduce pesticide waste and environmental pollution, improve the effectiveness of pesticide use, and thus, reduce pesticide inputs. Further, farmers will be more inclined to choose pesticides that are affordable and effective through ASS training and guidance. The choice of pesticides may be based on market recommendations and word-of-mouth, rather than relying entirely on specialized agricultural technical services. This choice may be affected by the price of pesticides, the economic conditions of farmers, and the competition in the pesticide market, among other factors. This precision in pesticide selection will largely improve the standardization of pesticide use to reduce pesticide inputs.

**Table 7:** Regression results on the size of farmland operations.

Matching methods	Large area group			Small area group		
	Treated	Controls	ATT	Treated	Controls	ATT
Nearest neighbour matching	5.793	4.250	1.543***(0.264)	4.790	3.160	1.630***(0.200)
Caliper matching	5.793	4.026	1.767***(0.237)	4.790	3.079	1.711***(0.170)
Spline matching	5.357	4.185	1.608***(0.229)	4.790	3.135	1.655***(0.146)
Kernel matching	5.793	4.138	1.655***(0.246)	4.790	3.124	1.666***(0.173)

***, **, and * indicate signiffcance at the 1%, 5%, and 10% levels, respectively.

#### Participation in cooperatives

Next, sample households are grouped based on whether they participate in cooperatives. Table 8 shows that the role of ASS in reducing pesticide inputs is greater for farm households participating in cooperatives across the four matches than for the households not participating in cooperatives. This is because cooperatives usually provide training and technical support to farmers to help them acquire the knowledge and skills to properly use pesticide. Further, the centralized purchasing of pesticides usually requires farmers to follow certain standards and specifications, including the type, dosage, and timing of pesticide use. This helps farmers better understand and use pesticides, and improves the effectiveness and safety of pesticide use. Meanwhile, farmers who participate in cooperatives usually pay more attention to environmental protection and sustainable development, and are more willing to adopt environmentally friendly agricultural technologies and measures. This in turn can also improve the efficiency of pesticide use and reduces pesticide inputs.

**Table 8:** Participation in cooperative regression analysis.

Matching methods	Participation in cooperatives			Not participation in cooperatives		
	Treated	Controls	ATT	Treated	Controls	ATT
Nearest neighbour matching	5.357	3.424	1.933***(0.361)	5.417	3.854	1.563***(0.202)
Caliper matching	5.357	3.443	1.913***(0.313)	5.417	3.695	1.721***(0.172)
Spline matching	5.357	3.607	1.750***(0.337)	5.417	3.803	1.614***(0.100)
Kernel matching	5.357	3.397	1.960***(0.323)	5.417	3.814	1.603***(0.190)

***, **, and * indicate signiffcance at the 1%, 5%, and 10% levels, respectively.

#### Non-farm employment

Next, the sample households are grouped whether they have members who have non-farm employment. From Table 9, the agricultural labor force may decrease as farmers shift to non-farm employment. This may lead to poor farmland management, and loss of agricultural knowledge and skills. Further, some farmers may ignore the long-term impacts of pesticide use in pursuit of short-term benefits, and thus, increase pesticide inputs. As such, the role of ASS in reducing pesticide use for non-farm employed households is smaller than that of those who are not. This is because households without non-farm employment practice intensive farming through measures such as manual weeding and better field management. Together with the promotion of ASS, this can have a greater effect on reducing pesticide use than non-farm employed farmers.

**Table 9:** Results of regressions with and without non-farm employment.

Matching methods	Non-farm employment			No non-farm employment		
	Treated	Controls	ATT	Treated	Controls	ATT
Nearest neighbour matching	5.292	3.746	1.545***(0.264)	5.478	3.881	1.597***(0.228)
Caliper matching	5.292	3.611	1.681***(0.238)	5.478	3.657	1.821***(0.197)
Spline matching	5.292	3.696	1.596***(0.260)	5.478	3.834	1.643***(0.202)
Kernel matching	5.292	3.676	1.616***(0.242)	5.417	3.814	1.603***(0.190)

***, **, and * indicate signiffcance at the 1%, 5%, and 10% levels, respectively.

### Mechanisms analysis

#### Green perception effect

Table 10 shows that ASS can promote the formation of environmentally friendly pesticide use habits by formulating service specifications and guiding farmers to adopt environmentally friendly behaviors. With the improvement of farmers’ environmental awareness, and the combination of their own environmental awareness and service guidance, they are more likely to follow the service specifications and reduce the irrational pesticide use behavior. In addition, within the village, farmers interact with each other and may share planting habits, especially under the specialized guidance provided by ASS. This can help increase the green perception of farmers, and thus, their adoption of new pesticides and green technologies. In turn, this can improve the efficiency of pesticide use, and thus, reduce pesticide inputs. Therefore, the green perception effect of ASS plays a positive and significant role in reducing pesticide inputs. Hence, hypothesis H1 is supported.

**Table 10:** Mechanism test regression results.

Variables	Tobit			
	Coeff	Std.	Coeff	Std.
Perceived × ASS	1.486***	0.050		
Demonstration × ASS			0.368***	0.025
Other Variables	Yes	Yes	Yes	Yes
_cons	8.526***	0.619	9.398***	0.693
*R*^2^	0.199		0.148	
*N*	2948		2948	

***, **, and * indicate signiffcance at the 1%, 5%, and 10% levels, respectively.

#### Demonstration-led effect

Table 10 shows that the demonstration-led effect of ASS plays a positive and significant role in reducing pesticide inputs. In remote rural areas, ASS is a new type of service. Hence, most farmers have a wait-and-see attitude. Meanwhile, ASS organizations will provide farmers with agricultural skills training and guidance, and popularize agricultural and environmental protection knowledge. As the number of farmers who purchase the service increases, the coverage of such training and guidance may be wider, thus helping more farmers master correct pesticide use and reduce irrational pesticide use. Moreover, ASS may promote collective action among farmers. The effect of such collective action may be more obvious when the number of driven farmers increases, thus improving the effectiveness of pest control and reducing pesticide use. Thus, hypothesis H2 is supported.

## Discussion

Pesticides, as new chemical agricultural materials, play an important role in increasing crop yields and controlling pests and diseases^37^. However, excessive pesticide use has resulted in high concentrations and toxicity of pesticide pollutants remaining in farmland, thereby aggravating agricultural surface pollution, lowering the quality of agricultural products, and jeopardizing human health^38^. Farmers’ behavior and motivation can directly determine the effect of pesticide use due to constrains in many aspects such as capital, technology, land, and labor^39^. With such poor long-term agricultural production practice, it has produced the problem of high pesticide use and low efficiency^40^. Therefore, realizing the goal of pesticide reduction must not be cut from the perspective of farmers. Existing research shows that factors affecting pesticide reduction include household characteristics, farmland quality, crop structure, pesticide type, and related policies^41–43^. However, in the study of pesticide use reduction, research is more inclined to farmland scale management perspective. The scarcity of farmland resources can induce farmers to use chemical agricultural materials to improve the marginal use efficiency of farmland. Meanwhile, with larger farms, farmers can effectively use agricultural machinery and quantify the pesticide use^44^. However, the pesticide reduction pathway for large-scale farmland management is constrained by land fragmentation and farmland quality. In China, the small amount of farmland per capita and relatively low quality of farmland have induced farmers to rely more on pesticides.

The development of ASS will help to promote the green transformation of agricultural production. Compared to farmers, ASS providers have advantages in technology, equipment and capital, and can rely on their own scale, and leverage the advantages of organization in terms of land and pesticide resources to effectively tackle crop pests and diseases through unified prevention and control^45^. This can also help standardizing pesticide use and improve its efficiency of use. ASS can effectively solve the problems of lack of family labor and insufficient supply of technology, promote the efficient allocation of agricultural resources, and improve the productivity of agricultural technology^46^. Meanwhile, agricultural machinery and equipment can be leased to farmers or contracted for pest control, effectively reducing the amount of pesticides through precise application of medicines, and united distribution and control, thus saving labor costs. In addition, ASS increase the green added value of agricultural products on the production side, and increase consumer trust in green agricultural products on the consumption side. Thus, farmers may have more incentives to adopt pesticide reduction technology to meet both the mandatory regulations and consumer demand^47,48^. Therefore, this study examines the green perception and demonstration effects of ASS to study the use of pesticide reduction, providing valuable reference and insights for future research and stakeholders.

## Conclusions and policy implications

### Conclusions

Using data from the 2020 CRRS, this study analyzes the impact of ASS on reducing pesticide inputs. The results are summarized as follows: (1) ASS play a significantly positive role in reducing pesticide inputs. Among influencing factors, the household head’s education level, farmland size, land parcel number, labor time, and distance from the town play significantly positive roles in reducing the amount of pesticide inputs. Total household income, pesticide packaging, pesticide hazards, package recycling, perceptions of application per mu, and village terrain play significantly negative roles in reducing pesticide inputs and are not conducive for reducing pesticide use. (2) Heterogeneity analysis shows that ASS were more effective in reducing pesticide inputs in the small area group, the participating cooperative group, and the no off-farm employment group than in the large area group, the non-participating cooperative group, and the non-farm employment group. (3) The test of the mechanism shows that: the ASS can effectively increase the green perceptions of the farm households, and also play a demonstration role for the farm households, which can help to reduce the pesticide inputs.

### Policy implications

First, the government should further promote ASS and publicity training. In particular, it should train and make farmers aware about the adverse consequences of the wrong application of various pesticides and their related knowledge of pharmacology, especially on the scientific application of pesticides. Further, the government must support the introduction of technology and equipment into agricultural production, and guide farmers to adopt pesticide reduction behaviors. Thus, the government should encourage and support farmers to adopt ASS while lowering their threshold to purchase it.

Second, efforts should be made to improve pesticide use efficiency by promoting continuous operation. In particular, the government should highlight the important power of village collectives, leverage their intermediary service advantages, and promote the effective "centralized farmland transfer + pesticide application trusteeship" model. Moreover, policy support for different types of ASS should be different according to farmers’ actual needs, local conditions, and the appropriate time to promote ASS.

Finally, farmers should be actively encouraged to participate in ASS. With the transfer of rural labor to the non-farming sector, agricultural cooperatives have become an organizational vehicle for providing socialized services. This can not only help solve the labor shortage caused by the transfer of farm labor, but also improve agricultural resource use efficiency. The government should also actively guide farm households to participate in ASS. Furthermore, the government should promote pest control services for different target groups, especially prioritizing those who are older and have smaller cropland areas. Together, this can promote smallholder production and help in achieving agricultural modernization.

## Data Availability

The data that support the findings of this study are available from China Rural Revitalization Survey but restrictions apply to the availability of these data, which were used under license for the current study, and so are not publicly available. Data are however available from the authors upon reasonable request and with permission of China Rural Revitalization Survey.
